# (*S*)-2-(Cyclobutylamino)-*N*-(3-(3,4-dihydroisoquinolin-2(1*H*)-yl)-2-hydroxypropyl)isonicotinamide Attenuates RANKL-Induced Osteoclast Differentiation by Inhibiting NF-κB Nuclear Translocation

**DOI:** 10.3390/ijms24054327

**Published:** 2023-02-21

**Authors:** Mina Ding, Eunjin Cho, Zhihao Chen, Sang-Wook Park, Tae-Hoon Lee

**Affiliations:** 1 BioMedical Sciences Graduate Program (BMSGP), Chonnam National University, Gwangju 61186, Republic of Korea; 2 Department of Oral Biochemistry, Dental Science Research Institute, School of Dentistry, Chonnam National University, Gwangju 61186, Republic of Korea

**Keywords:** osteoporosis, osteoclast differentiation, PRMT5, NF-κB, methylation

## Abstract

Osteoporosis is a common skeletal disease; however, effective pharmacological treatments still need to be discovered. This study aimed to identify new drug candidates for the treatment of osteoporosis. Here, we investigated the effect of EPZ compounds, protein arginine methyltransferase 5 (PRMT5) inhibitors, on RANKL-induced osteoclast differentiation via molecular mechanisms by in vitro experiments. EPZ015866 attenuated RANKL-induced osteoclast differentiation, and its inhibitory effect was more significant than EPZ015666. EPZ015866 suppressed the F-actin ring formation and bone resorption during osteoclastogenesis. In addition, EPZ015866 significantly decreased the protein expression of Cathepsin K, NFATc1, and PU.1 compared with the EPZ015666 group. Both EPZ compounds inhibited the nuclear translocation of NF-κB by inhibiting the dimethylation of the p65 subunit, which eventually prevented osteoclast differentiation and bone resorption. Hence, EPZ015866 may be a potential drug candidate for the treatment of osteoporosis.

## 1. Introduction

Osteoporosis is a common skeletal disease that occurs when the bone mineral density (BMD) decreases or the bone structure deteriorates [1]. The development of osteoporosis is primarily caused by the imbalance of bone homeostasis, including bone resorption by osteoclasts and bone formation by osteoblasts [2]. Post-menopause, old age, medications, endocrine disorders, immobilization, inflammatory arthropathy, hematopoietic disorders, and nutrition disorders increase osteoclast activity, leading to osteoporosis [3]. Given that the population’s average age is rapidly increasing worldwide, osteoporosis could become a major health concern that significantly impacts the quality of life of older adults [4]. Currently, the preferred treatment for osteoporosis is pharmacological interventions. Bisphosphonates, denosumab, and strontium ranelate are the main medicines for the treatment of osteoporosis [5]. However, some studies have shown a rapid decrease in BMD and an increased risk of vertebral fractures after the discontinuation of denosumab [6,7]. Additionally, there have been some reported side effects of bisphosphonates in the treatment of osteoporosis [8]. Therefore, this study aimed to find a new pharmacological agent to treat osteoporosis.

Osteoclasts are giant multinucleated cells that have critical roles in the regulation of bone development and bone homeostasis [9]. Osteoclasts are derived from cells of the monocyte/macrophage lineage by the activation of receptors by two factors, the macrophage-colony stimulating factor (M-CSF) and the receptor activator of nuclear factor-kappa B ligand (RANKL). M-CSF primarily regulates the proliferation and survival of osteoclast precursors and mature cells [10]. RANKL is the major osteoclast differentiation factor, and its interaction with RANK recruits tumor necrosis factor receptor-related factors and activates downstream signaling pathways, thereby inducing the nuclear factor of activated T cells 1 (NFATc1) [11,12]. NFATc1 has a major role in regulating several osteoclast-specific genes including matrix metallopeptidase 9 (*Mmp9*), Cathepsin K (*Ctsk*), and acid phosphatase 5, tartrate resistant (*Acp5*) [13,14].

Histone methylation is the modification of certain amino acids in histones, such as lysine, arginine, and histidine, by the addition of one to three methyl groups. Histone methylation is a dynamic process, and methyl groups can be added or removed by histone methyltransferases and histone demethylases [15,16]. These enzymes have been shown to be involved in tumorigenesis [17], angiogenesis [18], and the development of acute myeloid leukemia (AML) [19]. Studies have shown that histone methylation is regulated in bone cell differentiation [20]. The protein arginine N-methyltransferase (PRMT) family is a group of methyltransferases. There are two types of PRMTs: PRMT1, 3, 4, 6, and 8 are type I PRMTs that asymmetrically demethylate arginine, while PRMT5 and PRMT7 are type II PRMTs that symmetrically demethylate arginine [21,22]. PRMT5 is known to play important roles in gene transcriptional regulation and signal transduction [23,24]. Previous research has indicated that PRMT5 protein increases during osteoclastogenesis, and the reduction of PRMT5 via (*S*)-*N*-(3-(3,4-Dihydroisoquinolin-2(1*H*)-yl)-2-hydroxypropyl)-6-(oxetan-3-ylamino)pyrimidine-4-carboxamide (EPZ015666) that inhibits RANKL induced osteoclast differentiation [25]. (*S*)-2-(Cyclobutylamino)-*N*-(3-(3,4-dihydroisoquinolin-2(1*H*)-yl)-2-hydroxypropyl)isonicotinamide (EPZ015866) is another PRMT5 specific inhibitor, which blocks the enzyme activity of PRMT5 in the proliferation and cell cycle progression of human colorectal cancer cells [26]. This study investigates the underlying molecular mechanisms of EPZ015866 on osteoclast differentiation. 

Nuclear factor-κB (NF-κB) is a transcription factor that has an important role in the survival, formation, and functions of osteoclasts [27,28]. The inhibition of NF-κB has been shown to be an efficient method to suppress osteoclast formation and bone resorption [29,30]. Therefore, many studies have focused on NF-κB as a target for the treatment of osteoporosis [28,31]. Studies have confirmed that methylation of lysine and arginine residues in the p65 subunit of NF-κB regulates its activity [32,33]. Additionally, there is evidence that PRMT5 can regulate NF-κB activity through the methylation of p65 [34,35]. The present study demonstrates that EPZ015866, a derivative of EPZ015666, has a better therapeutic effect on osteoporosis than EPZ015666. 

## 2. Results

### 2.1. EPZ Compounds Attenuates RANKL-Induced Osteoclast Differentiation In Vitro

**Figure 1:**
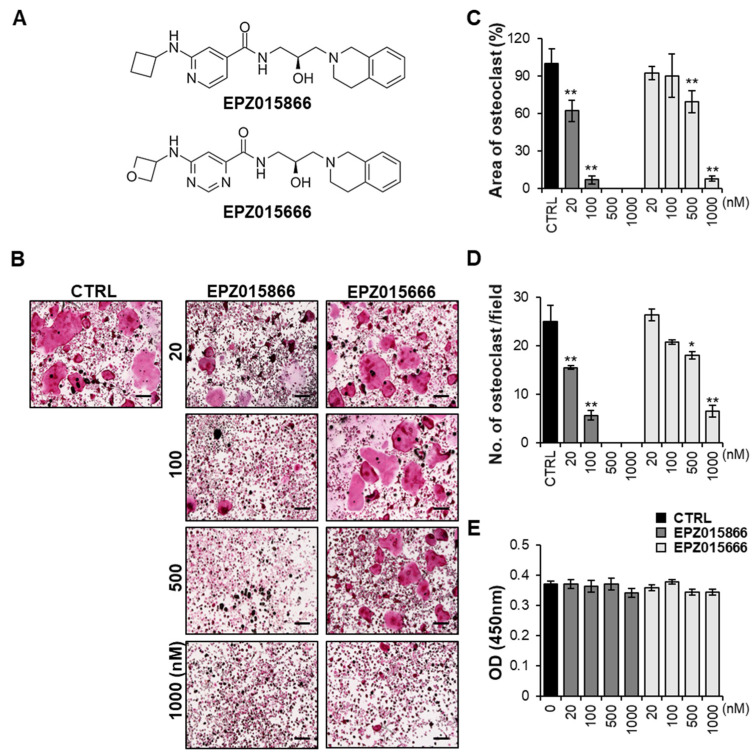
EPZ compounds attenuated RANKL-induced osteoclast differentiation. (**A**) The chemical structures of EPZ015866 and EPZ015666. (**B**) BMMs were treated with or without the indicated concentrations of EPZ compounds (EPZ015866 and EPZ015666), followed by M-CSF (30 ng/mL) and RANKL (50 ng/mL) stimulation for 4 days. Cells were fixed, and a TRAP staining assay was performed. The scale bar represents 200 µm. (**C**,**D**) Quantitative analysis of the area and the number of osteoclasts after treatment with the EPZ compounds was done using Image J software. (**E**) Cell viability was examined on day 2 after the stimulation with the EPZ compounds. “CTRL” indicates the control group (cells were treated with M-CSF and RANKL). Data are presented as the mean ± standard deviation (SD) of three independent experiments. * *p* < 0.05, ** *p* < 0.01 versus the control group.

EPZ015666 is a known PRMT5 inhibitor that suppresses osteoclast differentiation [25]. Since there is a structural similarity (Figure 1A), we compared EPZ015866 with EPZ015666 in a dose-dependent manner to investigate the effect of EPZ015866 as an inhibitor of osteoclast formation. Bone marrow-derived macrophages (BMMs) isolated from the femur and tibia of a mouse were stimulated with RANKL and M-CSF in the absence or presence of EPZ015866 or EPZ015666 at the indicated concentrations for four days. EPZ015866 significantly reduced RANKL-induced tartrate-resistant acid phosphatase (TRAP) positive multinucleated giant cell formation at a low dose, 20 nM, whereas EZP015666 inhibited it at a high dose, 1000 nM (Figure 1B). When we calculated the area of TRAP-positive cells and the number of mature osteoclasts containing more than three nuclei, EPZ015866 dramatically decreased the area and number of osteoclasts at a concentration of 20 nM, the same concentration at which EZP015666 did not significantly work (Figure 1C,D). Therefore, we suggest that EPZ015866 is an effective compound for inhibiting osteoclastogenesis and is better than EPZ015666. Both EPZ compounds were not cytotoxic when the concentration of the compounds was equal to or less than 1000 nM (Figure 1E). These results suggest that the EPZ compounds suppress the RANKL-induced osteoclastogenesis without causing cytotoxicity. Bone remodeling is regulated by the homeostasis between osteoclasts and osteoblasts [36]. Therefore, we indicated whether the EPZ compounds affected osteoblast differentiation. The osteoblast differentiation was analyzed by alkaline phosphatase (ALP) staining after bone morphogenetic protein 2 (BMP2) stimulation. Neither EPZ compound affected BMP2-induced osteoblastogenesis compared with the control (Figure S1A,B). These results suggest that EPZ compounds only suppress osteoclast differentiation and have no effect on osteoblast formation.

### 2.2. EPZ Compounds Suppress F-Actin Ring Formation and Bone Resorption

**Figure 2:**
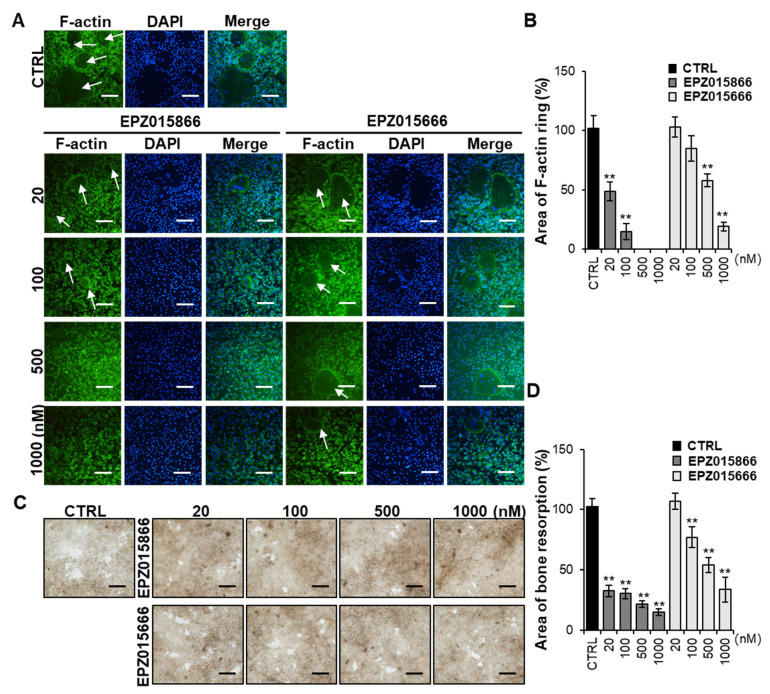
EPZ compounds suppressed F-actin ring formation and bone resorption. (**A**) Representative F-actin ring formation was analyzed. BMMs were stimulated by M-CSF and RANKL, followed by treatment with or without the EPZ compounds for 4 days. Cells were stained with Phalloidin and DAPI for the F-actin ring and nuclei, respectively. Scale bar = 100 μm. The arrow indicates the location of the F-actin ring. (**B**) The area of the F-actin ring was measured. (**C**) BMMs were differentiated into osteoclasts by treatment with or without the EPZ compounds for 5 days. The bone pit area was detected as empty white spots. Scale bar = 100 μm. (**D**) The area of the resorption pits was measured using Image J software. “CTRL” indicates the control group (cells were treated with M-CSF and RANKL). The data presented are the mean ± SD of three independent experiments. ** *p* < 0.01 versus the control group.

To determine whether the EPZ compounds inhibited F-actin ring formation, BMMs were treated with or without the EPZ compounds. F-actin ring formation was observed on day 4 after RANKL stimulation in the control group (Figure 2A). However, treatment with EPZ015866 remarkably reduced the size of F-actin ring structures, starting at 20 nM in a dose-dependent manner. Although the EPZ015666 treatment suppressed F-actin ring structures at high doses (500–1000 nM), it did not significantly inhibit them at low doses (Figure 2B). Additionally, we confirmed whether the EPZ compounds suppressed the bone-resorbing activity of osteoclasts. In the control group, bone resorption pits were detected after RANKL stimulation. However, bone resorption pits were decreased by the EPZ015866 treatment starting at 20 nM (Figure 2C). The area of the bone resorption pits was quantified according to the bone resorption assay results (Figure 2D). In the EPZ015666 treatment group, the inhibitory effect of the resorption pits was weaker than EPZ015866. These data indicated that EPZ015866 suppressed the formation of mature osteoclasts and the bone resorption ability better than EPZ015666.

### 2.3. EPZ Compounds Inhibit the Expression of Osteoclast-Specific Genes

**Figure 3:**
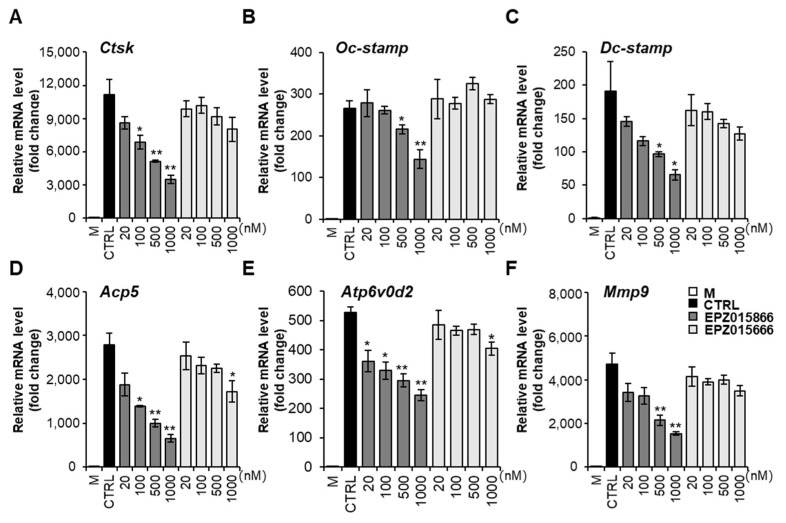
EPZ compounds suppressed the gene expression of osteoclastogenesis-related markers. BMMs were incubated with M-CSF and RANKL and treated with or without the indicated concentrations of the EPZ compounds for 4 days. The total mRNA was extracted, and the osteoclastogenesis-associated genes, such as *Ctsk* (**A**), *Oc-stamp* (**B**), *Dc-stamp* (**C**)*, Acp5* (**D**), *Atp6v0d2* (**E**), and *Mmp9* (**F**) were determined by RT-PCR. All the results were normalized to GAPDH. “M” indicates M-CSF, and “CTRL” indicates the control group (cells were treated with M-CSF and RANKL). The data presented are the mean ± SD of three independent experiments. * *p* < 0.05, ** *p* < 0.01 versus the control group.

To investigate the effect of the EPZ compounds on osteoclastogenesis-associated gene expression, mRNA expression levels were examined by RT-PCR. We found that the mRNA expression of osteoclast-specific genes, including *Acp5*, *Ctsk*, Dendritic cell-specific transmembrane protein *(Dc-stamp)*, Osteoclast stimulatory transmembrane protein *(Oc-stamp)*, *Atp6v0d2*, and *Mmp9* were suppressed in a dose-dependent manner by EPZ015866 (Figure 3A–F). However, EPZ015666 only significantly inhibited *Acp5* and *Atp6v0d2* expression at 1000 nM. These data reveal that EPZ015866 prevents osteoclast differentiation via inhibiting the expression of osteoclast-mediated genes better than EPZ015666 in vitro.

### 2.4. EPZ Compounds Decrease the Expression of the Transcription Factors PU.1 and NFATc1

**Figure 4:**
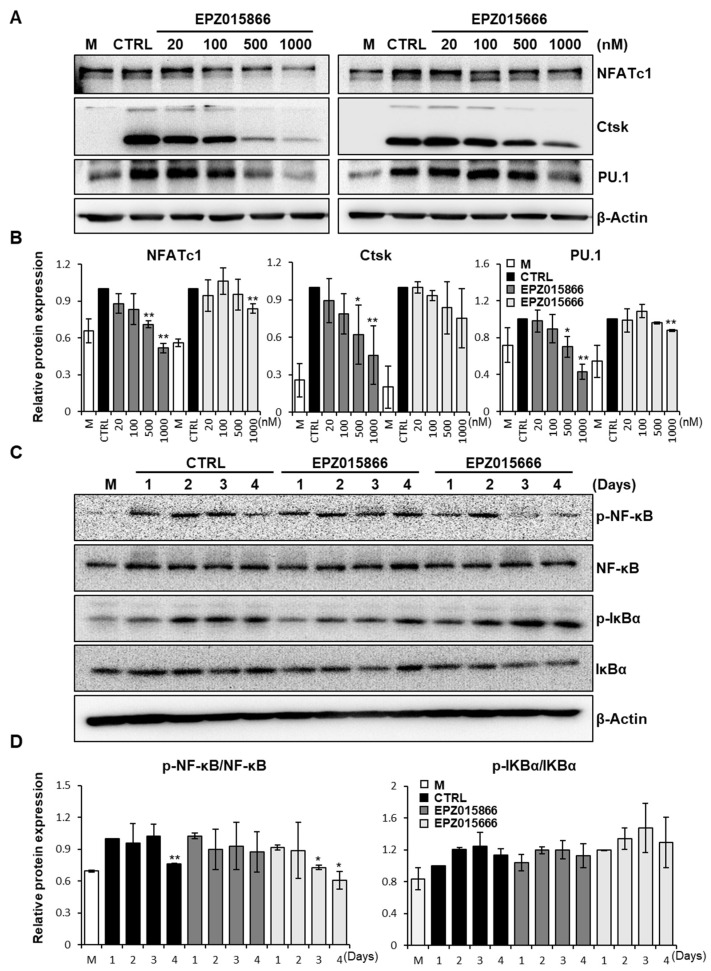
EPZ compounds attenuate osteoclast-related protein expression. (**A**) BMMs were differentiated into osteoclasts with or without EPZ compounds for 4 days. NFATc1, Ctsk, and PU.1 protein expression were examined for by Western blot. (**B**) The protein intensity was calculated by Image J, and calculated the ratio of the NFATc1, Ctsk, and PU.1 band intensity relative to the b-actin band. (**C**) BMMs were treated with or without EPZ compounds for the indicated days. p-NF-κB, NF-κB, p-IκBα, and IκBα were determined by Western blot. (**D**) The ratios of p-NF-κB, and p-IκBα band intensity relative to total NF-κB, and IκBα bands. The intensity of the bands were analyzed using Image J. All experiments were repeated three times. “M” indicates M-CSF, “CTRL” indicates the control group (cells were treated with M-CSF and RANKL). * *p* < 0.05, ** *p* < 0.01 versus the control group.

To demonstrate the molecular mechanisms by which the EPZ compounds may regulate osteoclastogenesis, we examined the expression levels of osteoclast-associated proteins. The protein levels of NFATc1, PU.1, and Ctsk were suppressed in a dose-dependent manner by EPZ015866 (Figure 4A,B). However, NFATc1 expression levels and PU.1 levels were decreased only at 1000 nM EPZ015666. The NF-κB signaling pathway plays a key role in osteoclast differentiation [37]. NF-κB expression levels were not significantly altered by EPZ compound treatment. However, p-NF-κB levels were slightly maintained by EPZ015866 until day 4, although its expression was decreased in the control and by EZP015666 (Figure 4C,D). As a regulator of NF-κB, p-IκBα and IκBα expression levels were not altered between the control and the EPZ compounds (Figure 4C,D). These data indicate that EPZ015866 suppresses osteoclast differentiation by reducing the transcription factors, PU.1 or NFATc1, and altering the expression of p-NF-κB. In addition, the NF-κB gene level in osteoclast was checked after treatment with the EPZ compounds by RT-PCR. The result suggests that the NF-κB gene is not regulated by the EPZ compounds (Figure S2A). Luciferase assay was performed on the NF-κB luciferase activity in the NF-κB reporter HEK293 cell line after treatment with the EPZ compounds. The result showed the NF-κB transcriptional activity was not altered by the EPZ compounds (Figure S2B). These results indicate that the NF-κB transcriptional is not regulated by the EPZ compounds.

### 2.5. EPZ Compounds Downregulate PRMT5 and H3R8me2s/H4R3me2s but Do Not Impact mRNA Levels of Prmt5 during Osteoclastogenesis

**Figure 5:**
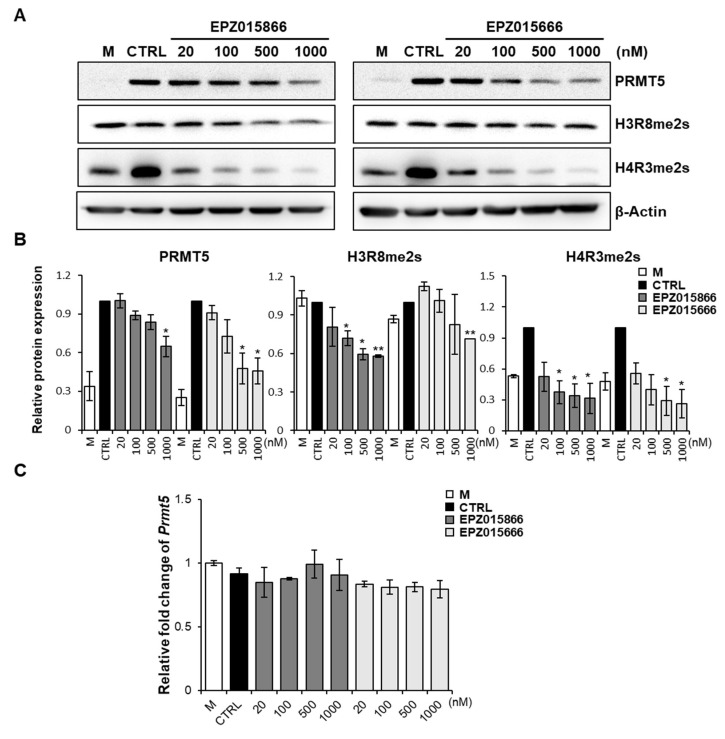
EPZ compounds downregulate PRMT5 expression and activation but do not *Prmt5* mRNA levels. (**A**) BMMs were treated with or without EPZ compounds in the presence of M-CSF (30 ng/mL) and RANKL (50 ng/mL) for 3 days. Western blot was performed to determine the protein expression of PRMT5, H4R3me2s, and H3R8me2s. (**B**) The protein intensity was calculated by Image J, and calculated the ratio of the PRMT5, H4R3me2s, and H3R8me2s band intensity relative to the b-actin band. * *p* < 0.05, ** *p* < 0.01 versus the control group. (**C**) RT-PCR assay was performed to test the mRNA expression levels of *Prmt5* in the control or EPZ compounds-treated cells. The data presented are the mean ± SD of three independent experiments. “M” indicates M-CSF, “CTRL” indicates the control group (cells were treated with M-CSF and RANKL).

To confirm whether the expression and activity of PRMT5 were regulated by the EPZ compounds during RANKL-induced osteoclastogenesis, BMMs were treated with various concentrations of the EPZ compounds (Figure 5). Because PRMT5 regulates the dimethylation of arginine symmetrically [23], we examined the symmetric demethylation of histone H3 arginine 8 (H3R8me2s) and the symmetric demethylation of histone H4 arginine 3 (H4R3me2s) levels to determine the activity of PRMT5 (Figure 5A,B). PRMT5 expression was suppressed in a dose-dependent manner in the cells treated with EPZ015866 or EPZ015666 compared with the control group. Furthermore, the methylation level of H4R3me2s was significantly increased when stimulated with RANKL in the control, but its expression was significantly inhibited in EPZ015866- or EPZ015666-treated groups. However, the methylation level of H3R8me2s was only suppressed by EPZ015866. Interestingly, the *Prmt5* mRNA levels were not altered during osteoclastogenesis by the EPZ compounds (Figure 5C). These data indicate that the EPZ compounds suppress PRMT5 expression and suggest their activity in RANKL-induced osteoclast differentiation.

### 2.6. EPZ Compounds Reduce NF-κB Nuclear Translocation by Blocking the Demethylation of p65

**Figure 6:**
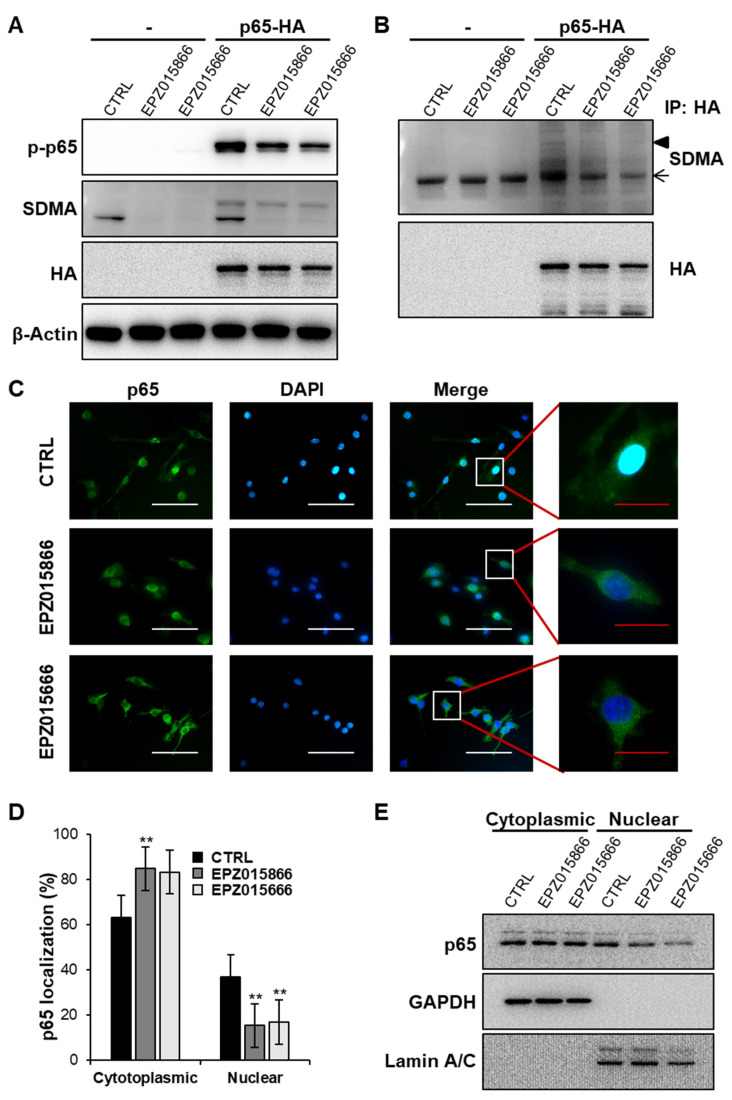
EPZ compounds suppress NF-κB translocation in osteoclasts. (**A**) Raw 264.7 cells and pcDNA3-HA-p65 (HA-p65) transfected Raw 264.7 cells were treated with RANKL and EPZ compounds for 48 h, and then assessed by Western blot. (**B**) Raw 264.7 cells and pcDNA3-HA-p65 transfected Raw 264.7 cells were treated with RANKL and EPZ compounds for 24 h for IP. IP was performed with anti-HA antibodies, followed by blotting with anti-SDMA or -HA antibodies. An arrowhead indicates the p65-HA band, and an arrow indicates non-specific immuno-globulins. (**C**) Immunofluorescence staining was also performed to detect the nuclear translocation of NF-κB. White scale bar = 100 μm. Red scale bar = 25 μm. (**D**) The graph represents the number of cells expressing p65 in the cytoplasmic or nuclear in C. A total of 10 slides were examined to calculate the numbers. ** *p* < 0.01 versus the control group. (**E**) The nuclear and cytoplasmic extraction were confirmed by the subcellular expression of p65. “CTRL” indicates control group (cells were treated with M-CSF and RANKL).

To demonstrate PRMT5 activation of NF-κB via the demethylation of the p65 subunit of NF-κB [32], we examined the expression levels of symmetric dimethylarginine (SDMA) in Raw 264.7 cells transfected with a pcDNA3-HA-p65 plasmid vector (Figure 6A). p-p65 expression was suppressed by the EPZ compounds compared to the control. Although non-transfected Raw 264.7 cells expressed p-p65 in response to RANKL stimulation, the expression levels were lower than in the transfected cells. Interestingly, SDMA levels were slightly reduced by the EPZ compounds even though we detected whole SDMA levels. To examine whether the EPZ compounds regulate the activity of p65 by blocking SDMA, immunoprecipitation (IP) was performed with anti-HA−Agarose antibody and then analyzed with anti-SDMA antibodies (Figure 6B). The data show that the SDMA of p65 had a high expression in the control group, which was reduced after EPZ compound treatment. To further investigate the role of methylation in the NF-κB pathway, we examined the translocation of NF-κB. Interestingly, the EPZ compounds inhibited the p65 expression in the nucleus compared to the control. Immunofluorescence was performed to investigate the nuclear translocation of p65 (Figure 6C). p65 was detected in the nucleus by RANKL stimulation on day 2 in control; however, the EPZ compounds effectively inhibited the nuclear translocation of p65. The quantitative analysis results of Figure 6C were shown in Figure 6D. The number of p65 in the cell nucleus was significantly decreased in the EPZ compound treatment groups compared with the control. The nuclear and cytoplasmic fractionation were also separated after RANKL stimulation to determine the expression levels of p65 (Figure 6E). Although nuclear p65 levels were decreased by the EPZ compounds, p65 levels in the cytoplasm were not significantly altered. These results indicate that the inhibitory effect of EPZ015866 on osteoclast differentiation is mediated by reducing the nuclear translocation of NF-κB. These results also indicate that the EPZ compounds inhibit the nuclear translocation of NF-κB by blocking the dimethylation of p65. 

## 3. Discussion

In the present study, we demonstrated that EPZ015866 and EPZ015666, known inhibitors of PRMT5, inhibit osteoclast differentiation as promising anti-osteoclastogenesis agents. The inhibition of osteoclastogenesis by EPZ015666 has previously been studied [25], but our current study found a more effective compound, EPZ015866, that could be used at a lower concentration in osteoclastogenesis. Our results indicated that, in the same concentration, EPZ015866 had a more significant inhibitory effect on osteoclast differentiation than EPZ01566 (Figure 1). Based on the area of TRAP-positive cells, the half maximal inhibitory concentration (IC_50_) values of EPZ015866 and EPZ015666 were around 30 nM and 600 nM, respectively. EPZ015866 reduced RANKL-induced osteoclast differentiation significantly better than EPZ015666 in vitro.

Mature osteoclasts firmly attach themselves to the bone surface using specialized actin rings through cytoskeletal reorganization and cell polarization, ultimately leading to bone resorption [9]. The F-actin ring indicates the fusion state of osteoclasts and is required for osteoclast formation and activation [38]. It has been reported that actin ring formation is a structural factor essential for bone resorption [39]. In our study, EPZ015866 and EPZ015666 inhibited the formation of actin rings in a dose-dependent manner, resulting in reduced formation of mature osteoclasts and marked inhibition of bone resorption.

Osteoclast differentiation is controlled by the transcriptional activation or repression of target genes by transcription factors. NFATc1 and PU.1 play important roles as transcriptional activators in osteoclast differentiation [40,41]. NFATc1 is a major regulator of osteoclast differentiation [42]. NFATc1 regulates the transcription of osteoclast-specific markers, including *Acp5*, *Atp6v0d2*, and *Ctsk*, which are important for the activation of mature osteoclasts [43]. We observed that *Acp5*, *Ctsk*, *Oc-stamp*, *Dc-stamp*, *Atp6v0d2*, and *Mmp9* mRNA expression levels were significantly inhibited in EPZ015866 treatment, although only *Acp5* and *Atp6v0d2* levels were reduced at high doses of EPZ015666 (Figure 3). EPZ015866 showed more effective suppression via inhibiting all osteoclast-associated genes at low doses. Some studies have shown that the expression of osteoclast-specific markers is regulated by the transcription factors, NFATc1, PU.1, and NF-κB [44]. Moreover, PU.1 has been reported as a transcriptional activator of NFATc1 involved in the expression of osteoclast-specific genes, including *Ctsk*, *Acp5,* and *Itgb3* [45]. Our results demonstrated that EPZ015866 more effectively inhibited the protein expression of NFATc1 and PU.1 than EPZ015666, although their regulatory mechanism is unknown or indirect regulation by EPZ compounds. Moreover, there are p65 independent pathways [46], including the alternative NF-κB pathway and direct regulation of NF-κB subunits in osteoclastogenesis [47,48]. These combined results suggest that EPZ015866 suppresses osteoclastogenesis-related genes through the expression of transcription factors PU.1 and NFATc1.

PRMT5 catalyzes the symmetric dimethylation of histone proteins to induce gene silencing by generating repressive histone marks, including H3R8me2s and H4R3me2s [23]. Thus, we checked the expression and activity of PRMT5 during RANKL-induced osteoclast differentiation. Our results suggest that EPZ015866 and EPZ01566 suppress the activation of PRMT5 during osteoclastogenesis. Although EZP015666 did not greatly inhibit H3R8me2s, it did inhibit PRMT5 protein levels.

The NF-κB signaling pathway plays an important role in RANKL-stimulated osteoclast differentiation [37,49]. RANKL-mediated NF-κB activation is further transmitted by inducing the transcription factor NFATc1 in BMMs. Furthermore, during the regulation of NF-ĸB activation by the IκB kinase (IKK) complex, the NF-ĸB/Rel dimer proteins are themselves subject to complex regulation through a series of post-translational modification (PTM) events [50]. Numerous studies have confirmed that PTM on the p65 subunit of NF-κB includes methylation [34], acetylation [51], and ubiquitination [52]. Moreover, Levy. et al. confirmed that the methylation and phosphorylation of p65 are mutually regulated, forming a more complex NF-κB regulatory system [53]. In addition, previous research shows that NF-κB is activated by the dimethylation of arginine 30 of the p65 subunit [54]. PRMT5 regulates the methylation of the arginine residues of p65 [33]. Therefore, we investigated whether the EPZ compounds inhibited p65 methylation in osteoclasts. Interestingly, as shown in Figure 6, pcDNA3-HA-p65 transfected Raw 264.7 cells revealed decreased expression of whole SDMA levels via the EPZ compounds. Additionally, p65-specific SDMA levels were decreased in EPZ treatment, assessed by IP analysis, which is correlated with SDMA regulation by the EPZ compounds [55]. We observed whether a reduction in the SDMA of p65 regulates the subcellular localization of p65. The results of immunostaining and Western blot showed that RANKL stimulation enhanced the translocation of p65 into the nucleus, while the EPZ compounds attenuated NF-κB activation by interfering with the nuclear translocation of p65. Taken together, the EPZ compounds repress p65 nuclear translocation via the inhibition of p65 dimethylation, leading to the prevention of osteoclast formation. It has been studied that the PRMT5-mediated methylation of the p65 subunit of NF-κB at R30 is involved in the regulation of p65 activity [34]. Harris, D.P. et al. (2014) indicated that PRMT5 symmetrically methylates R30 and R35 of NF-κB/p65 in TNF-α-activated endothelial cells [35]. In different research, Harris, D.P. et al. (2016) also suggested that the PRMT5-mediated methylation of p65 at R174 is required for the induction of CXCL11 in TNF-α-activated endothelial cells [56]. Therefore, we speculate that the role of PRMT5 in the regulation of p65 activity might be by regulating the methylation of R174, R35, and R30 of the NF-κB/p65 subunit in osteoclast differentiation. We will continue to explore in more detail which methylation sites of p65 mediate the suppression of osteoclast differentiation by PRMT5 inhibitors in the future.

Duncan et al. compared the inhibitors of PRMT5 based on their structure and medicinal chemistry optimization [57]. In their results, EPZ015866 shows lower PRMT5 IC_50_ and lymphoma cell line proliferation IC_50_ values than EPZ015666, suggesting a greater inhibitory effect on PRMT5. Therefore, EPZ015866 inhibits at lower concentration than EPZ015666 in osteoclast differentiation. However, both EPZ compounds inhibited the methyl status of p65 at a high dose (1000 nM), suggesting that the final inhibitory effect on the substrate may be the same, although further study is necessary to prove their pharmacological inhibitory effect on the substrate.

**Figure 7:**
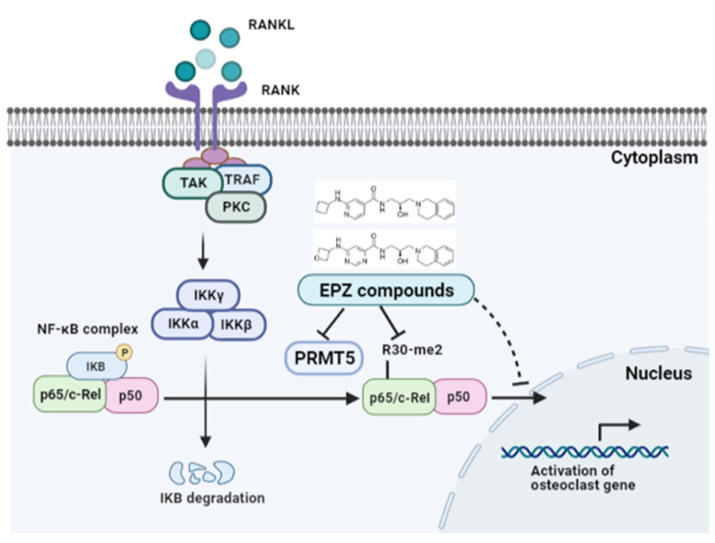
EPZ compounds inhibited osteoclast differentiation through the inhibition of NF-κB nuclear translocation, and arginine methyla-tion-mediated NF-κB activity. EPZ compounds indicates EPZ015866 and EPZ015666; TAK: TGFβ-activated kinase; TRAF: TNF receptor associated factors; PKC: Protein kinase C. IKK: IκB kinase (IkappaB kinase); IKB: Inhibitor of nuclear factor kappa B; P: Phos-phorylation; R30-me2: symmetric dimethylarginine of Arginine 30. This schematic diagram created with BioRender.com accessed on 13 February 2023.

In conclusion, the EPZ compounds significantly reduced RANKL-induced osteoclast differentiation, F-actin ring formation, and bone resorption. The EPZ compounds were identified as potent inhibitors of NF-κB activity and were shown to inhibit osteoclast differentiation through the inhibition of NF-κB nuclear translocation (Figure 7). Furthermore, we indicated that arginine methylation-mediated NF-κB activity has a critical role in RANKL-induced osteoclast differentiation. Therefore, these EPZ compounds may be suitable for drugs for treating bone diseases characterized by excessive osteoclast activity.

## 4. Materials and Methods

### 4.1. Materials and Reagents

The alpha modification of Eagle’s minimal essential medium (α-MEM) and fetal bovine serum (FBS) were purchased from Thermo Fisher Scientific (Waltham, MA, USA). Recombinant mouse M-CSF and RANKL were procured from Peprotech (Cranbury, NJ, USA). A tartrate-resistant acid phosphatase staining kit was bought from CosmoBio (Tokyo, Japan). Characterized fetal bovine serum (chFBS) was purchased from Hyclone (Logan, UT, USA). Alpha MEM (αMEM, without ascorbic acid) was purchased from Welgene (Taipei, Taiwan). Recombinant human BMP2 was provided by Sino biological (Wayne, PA, USA) and dissolved in distilled water. Phalloidin was bought from Thermos Fisher Scientific (Waltham, MA, USA). 4′,6-diamidino-2-phenylindole (DAPI) stain was purchased from Sigma–Aldrich (St. Louis, MO, USA). Specific antibodies for NFATc1 (#8032s), Ctsk (#48353), PU.1 (#2266), NF-κB (#4764s), p-NF-κB (#3033), IκBα(#9242s), and p-IκBα (#2859s), and secondary antibodies were all purchased from Cell Signaling Technology (Beverly, MA, USA). PRMT5 (ab109451) was purchased from Abcam (Cambridge, UK), and H3R8me2s (A2374) and H4R3me2s (A3159) were purchased from ABclonal Technology (Woburn, MA, USA). EPZ015866 (PubChem CID: 117072552) and EPZ015666 (PubChem CID: 90241673) were purchased from Chemscene and Selleckchem, respectively.

### 4.2. Osteoclast Cell Culture and Viability Assay

Mouse bone marrow cells were obtained from the femur and tibia of 10-week-old C57BL/6J mice, as described previously [58]. Briefly, red blood cells in bone marrow immune cells were lysed with Ammonium-Chloride-Potassium lysing buffer and then cultured in complete medium (α-MEM containing 10% FBS and 1% P/S) at the 37 °C in humidified air with 5% CO_2_ for 1 day. Non-adherent cells were harvested and cultured in Petri dishes for BMM selection with the complete medium in the presence of 30 ng/mL M-CSF. After three days, adhesion cells (BMMs) were harvested by Enzyme Free Cell Dissociation Solution Hank’s Based. The harvested cells were cultured further in the induction medium to induce the differentiation of osteoclasts.

For the cell viability study, BMMs were cultured at a density of 1 × 10^4^ cells per well in 96-well plates for 24 h. The cells were treated with M-CSF or M-CSF and RANKL (CTRL group) in the presence or absence of the indicated concentrations of EPZ compounds. After 48 h, the cell viability was assessed using an EZ-Cytox Kit. The experiment protocol was conducted following the manufacturer’s manual. Finally, the optical density was measured at 450 nm using a microplate reader (San Jose, CA, USA).

### 4.3. Tartrate-Resistant Acid Phosphatase (TRAP) Staining Assay

For the TRAP staining assay, a TRAP staining kit was obtained from Takara Biotechnology (Shiga, Japan). This kit was used in accordance with the manufacturer’s instructions. BMMs were cultured in 96-well plates in complete α-MEM containing 30 ng/mL M-CSF. After 24 h, the cells were treated with various concentrations of the EPZ compounds that were changed every two days during the experiment period. Afterward, the culture medium was replaced, and cells were fixed in 4% PFA at room temperature for 20 min and then stained for TRAP. Micrographs of cells were captured by the microscope, and the area of TRAP+ multinucleated osteoclasts (≥3 nuclei) was quantified using the Image J software (1.8.0_112 version, National Institutes of Health, Bethesda, MD, USA).

### 4.4. Osteoblast Cell Culture and In Vitro Differentiation

Primary calvarial cells were obtained from three-day-old mice by enzyme digestion. Briefly, the calvarias were cut into pieces and incubated in a digestion solution (0.1% type I collagenase with 0.2% Dispase II) at 37 °C for 40 min. After digestion, the calvarias were washed twice with a complete culture medium and cultured in a 10 cm dish at 37 °C in 5% CO_2_ for 3–4 days. Primary osteoblasts growing out of the bone chips were harvested and seeded into the 96-well plate for further experiments. For in vitro osteoblast differentiation, the cells were treated with or without the EPZ compounds in the presence of BMP2 (100 ng/mL) for seven days. Media were refreshed every two days. At the end of differentiation, cells were washed with PBS, fixed in 70% ice-cold ethanol for 30 min, and rinsed with distilled water two times. The cells were stained with BCIP^®^/NBT Liquid Substrate System for 20 min at room temperature. Images were captured with a microscope, and the intensity of ALP staining was quantified using the Image J software.

### 4.5. Phalloidin Staining and Immunofluorescence (IF) Staining

BMMs were seeded in 12-well plates at a density of 1.5 × 10^5^ per well. The EPZ compounds were added to the wells co-treated with RANKL for five days. After treatment, the cells were fixed with a 4% paraformaldehyde solution for 20 min at room temperature. The fixed cells were permeabilized with 0.1 % Triton-X 100 and blocked with 2 % BSA for 1 h. For immunofluorescence staining, the cells were incubated with a p65 primary antibody at 4 °C overnight. After the cells were washed with PBS, a goat anti-Mouse IgG (H+L) highly cross-adsorbed secondary antibody was incubated for 3 h at room temperature in the dark. After one day, the cells were washed with PBS. Finally, the cells were stained with 4′,6 diamidino2 phenylindole (DAPI). 

F-actin ring formation is a critical indicator of the bone resorption activity of osteoclasts and is a characteristic of mature cytoskeletal in osteoclasts [59]. Phalloidin staining was performed on osteoclasts treated with DMSO or EPZ compounds for four days, as described previously [60]. The cells were stained with FITC-conjugated Phalloidin for 45 min. After incubation with Phalloidin, the cells were washed with PBS. Finally, nuclei were visualized with DAPI. Images were captured by fluorescence microscopy.

### 4.6. Bone Resorption Assay

The effect of the EPZ compounds on bone resorption was assessed in accordance with the method of a previous study [61]. To explore the effect of the EPZ compounds on osteoclast-mediated bone resorption, BMMs were seeded into a 48-well bone resorption assay plate (2.5 × 10^4^ cells/well). After 24 h, the cells were treated with DMSO or the EPZ compounds, along with M-CSF and RANKL, for five days further. After cell differentiation, the attached cells were treated with 5% sodium hypochlorite for 5 min. The plates were air-dried at room temperature, and resorption pits were captured using a microscope. The total resorption area was quantified by Image J software.

### 4.7. Western Blot Assay

BMMs were seeded on six-well plates (3 × 10^5^/well) and cultured overnight. Then, the cells were stimulated with RANKL and treated with the indicated concentrations of the EPZ compounds. At the end of the differentiation, the cells were harvested using a plastic cell scraper and lysed with radioimmunoprecipitation assay (RIPA) buffer. The supernatant was collected following sonication and centrifugation. The concentration of proteins was detected by a BCA protein assay following the manufacturer’s protocol. The same amounts of protein (10 μg) were separated by polyacrylamide gel electrophoresis (PAGE) and transferred to polyvinylidene difluoride membranes (Bio-Rad Laboratories, Hercules, CA, USA). The membranes were blocked with 5% non-fat dry milk for 1 h at room temperature, and then incubated with primary antibodies at 4 °C overnight. The next day, the membranes were incubated for 2 h with secondary antibodies at room temperature, and signals were visualized by the enhanced chemiluminescence (ECL) western blotting detection reagent (Cytivalifescences, Marlborough, MA, USA).

### 4.8. Real-Time PCR Assay

Total RNA was extracted from cells with TRIzol reagent (Qiagen Sciences, Valencia, CA, USA), and then reversely transcribed using a PrimeScript RT Reagent Kit following the manufacturer’s instructions (Takara Bio-technology, Shiga, Japan). The cycling conditions were 37 °C for 30 min, 85 °C for 15 s, and storage 4 °C. Quantitative PCR was performed using a QuantStudio 3 real-time PCR system (Applied Biosystems, Foster City, CA, USA) with a Power SYBR Green PCR Master Mix. The mouse glyceraldehyde-3-phosphate dehydrogenase (GAPDH) gene was used as the control gene. The primers employed for the amplification are presented in Table S1.

### 4.9. Nuclear and Cytoplasmic Extraction

The cells’ nuclear and cytoplasmic proteins were extracted using the NE-PER Nuclear and Cytoplasmic Extraction Reagents (Thermo Scientific, Waltham, MA, USA) ac-cording to the manufacturer’s instructions. In brief, the cells were seeded in 10 cm dishes (1.5 × 10^6^ cells per dish). After one day, the cells were stimulated with RANKL and co-treated with EPZ015866 or EPZ015666 for 48 h. The cells were lysed with CER I and CER II buffer and centrifuged at 16,000× *g* for 5 min at 4 °C, and the supernatant (cytosolic protein) was stored at −80 °C. Nuclear pellets were re-suspended with NER buffer and vortexed to extract the nuclear protein, followed by incubation on ice for 40 min. The samples were then centrifuged at 16,000× *g* for 10 min at 4 °C. The supernatant (nuclear protein) was transferred to microtubes immediately after centrifugation. Finally, the nuclear and cytoplasmic proteins were analyzed by Western blot. 

### 4.10. Cell Transfection and Immunoprecipitation (IP)

Constructs were transfected into Raw264.7 cells using the NeonTM transfection system. The pcDNA3-HA-p65 plasmid DNA was a kind gift from Professor Park Jun Soo of the Division of Biological Science and Technology of Yonsei University. Raw 264.7 cells were transiently transfected with pcDNA3-HA-p65 plasmid DNA. The following day, the cells were cultured in complete media for an additional 48 h. 

Transfected Raw 264.7 cells were cultured with RANKL in the presence or absence of the indicated concentrations of EPZ compounds for two days. The cells were rinsed with PBS, harvested, and lysed in IP lysis buffer (20 mM Tris–HCl, pH 7.5, 150 mM NaCl, 10% glycerol, and 1% Triton X-100) containing protease inhibitors. The whole cell lysates were incubated on ice for 30 min, collected by centrifugation, and quantified. After the quantification of protein samples, equal volumes of protein samples were incubated with the Monoclonal Anti-HA−Agarose antibody at 4 °C overnight under gentle shaking. The resin was harvested by centrifugation, washed five times with PBS, and re-suspended in an equal volume of 2 × SDS loading buffer. All samples were heated at 95 °C for 5 min. Finally, the protein samples were separated by 10% SDS-PAGE. Western blot assays were performed as described previously.

### 4.11. Statistical Analysis

The results were presented as mean ± SD from three independent experiments in this study. The significant differences between the control and experimental groups were determined by a two-tailed Student’s *t*-test. Data were analyzed with GraphPad Prism 6.0 (GraphPad Software Inc., San Diego, CA, USA). * *p* < 0.05 and ** *p* <0.01 were considered significant.

## Data Availability

The data that support the findings of this study are available from the corresponding author upon reasonable request.

## Supplementary Materials

The following supporting information can be downloaded at: https://www.mdpi.com/article/10.3390/ijms24054327/s1.
