# Supplementary material for: (S)-2-(Cyclobutylamino)-N-(3-(3,4-dihydroisoquinolin-2(1H)-yl)-2-hydroxypropyl)isonicotinamide Attenuates RANKL-Induced Osteoclast Differentiation by Inhibiting NF-κB Nuclear Translocation
Source: Int J Mol Sci. 2023 Feb 21;24(5):4327. doi: 10.3390/ijms24054327 (PMC10002170; doi:10.3390/ijms24054327)
Supplement: Supplementary file 1 [file ijms-24-04327-s001.zip › ijms-2202551-supplementary.pdf]

International Journal of
Molecular Sciences

MDPI

Supplementary Materials

(*S*)-2-(Cyclobutylamino)-*N*-(3-(3,4-dihydroisoquinolin-2(1*H*)-yl)-2-hydroxypropyl)isonicotinamide attenuates RANKL-induced osteoclast differentiation by inhibiting NF-κB nuclear translocation

Supplementary results

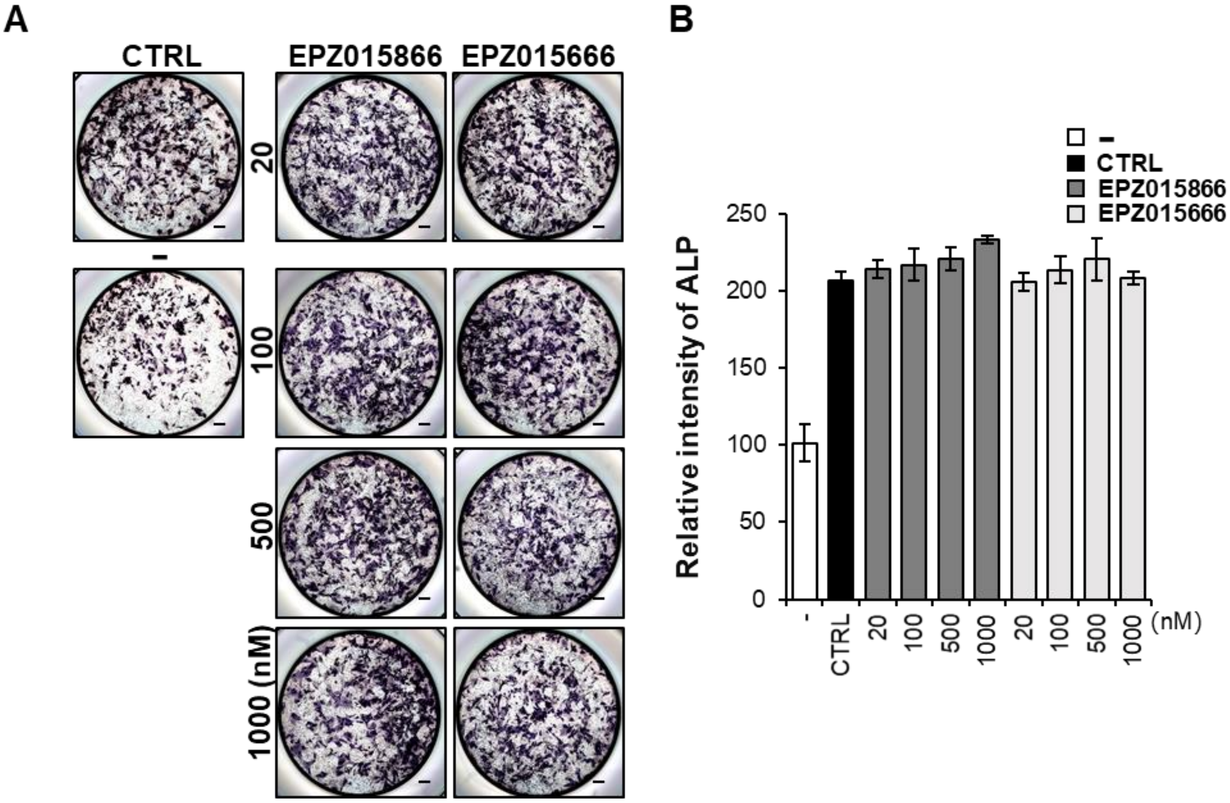

Figure S1. EPZ compounds did not affect the osteoblast formation. (**A**) Representative images of ALP staining showed mouse primary pre-osteoblast cells were cultured with BMP2 (100 ng/mL) in the presence or absence of different doses of EPZ compounds (20, 50, 100, 500, and 1000 nM) for 7 days. Scale bar represents 400 μm. (**B**) Quantified analysis of ALP staining intensity. "-" indicates cells were not treated with BMP2 and "CTRL" indicates the control group (cells were treated with BMP2).

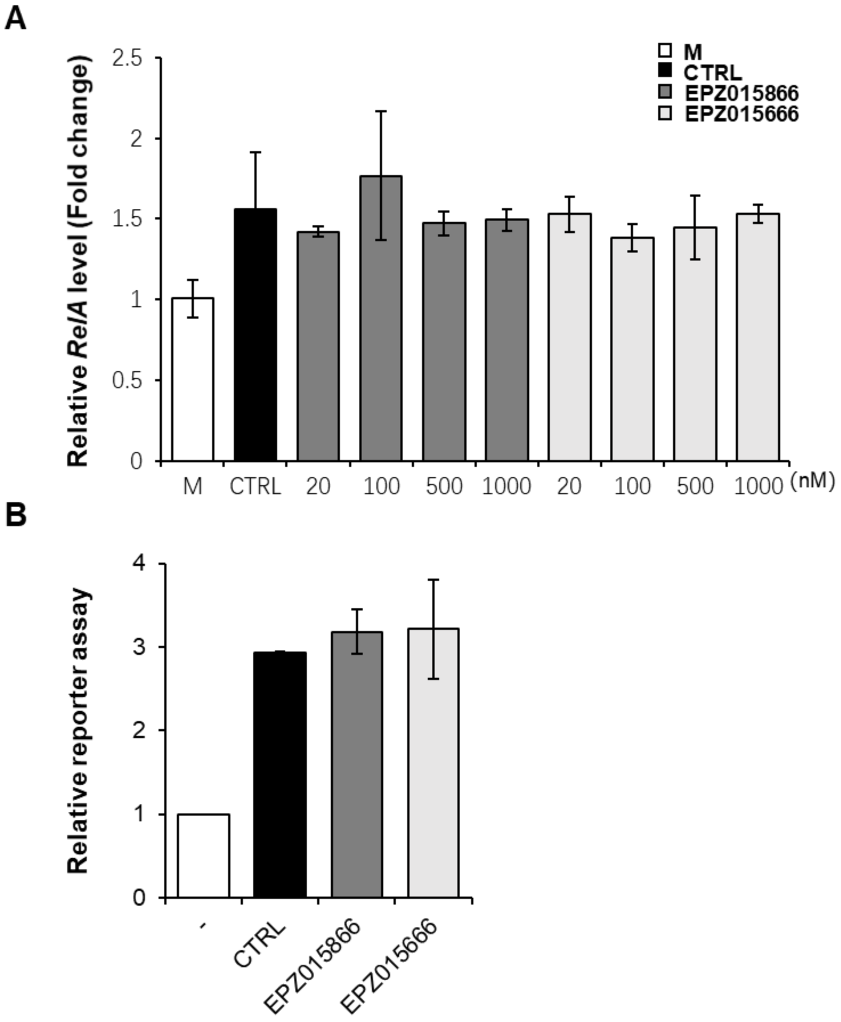

Figure S2. NF-κB transcriptional activity was not altered by EPZ compounds. (**A**) The mRNA levels of RelA (p65) in BMMs were determined by qRT-PCR after RANKL or indicated concentrations of EPZ compounds for 4 days. (**B**) The NF-κB reporter HEK293 cell line were incubated with or without 1000 nM of EPZ compounds for 24 hours and then stimulated with RANKL for 6 hours. NF-κB luciferase assay was performed. The data presented are the mean ± SD.

Table S1. Primer sequences used for real-time PCR analysis.

Gene (mouse)	Primer Sequence (5'-3')
Ctsk	F: ACTTCCGCAATCCTTACCGA<br>R: TTCGCTAGGCTCTTTTCGGA
Dc-stamp	F: CGCACGATGCTTCATTCTTC<br>R: CAGTGCCAGCCGCAATC
Oc-stamp	F: CAGAGTGACCACCTGAACAAACA<br>R: TGCCTGAGGTCCCTGTGACT
Acp5	F: TTTATGCTGGACACAGTGATGCT<br>R: CCCAGGTCTCGAGGCATT
Mmp9	F: CTGGACAGCCAGACACTAAAG<br>R: CTCGCGGCAAGTCTTCAGAG
Atp6v0d2	F: GTGAGACCTTGGAAGACCTGAAA<br>R: TCCTCATCTCCGTGTCAATTTTG
Prmt5	F: CTGAATTGCGTCCCCGAAATA<br>R: AGGTTCCTGAATGAACTCCCT

Gapdh	F: TGTGTCCGTCGTGGATCTGA<br>R: GATGCCTGCTTCACCACCTT

F: forward; R: reverse, Ctsk: cathepsin K.

Supplementary Method

NF-κB luciferase assay

The NF-κB reporter HEK293 cell lines were seeded into 12-well plates at a density of 1.0×10^5 per well. After 1 day, cells were incubated with or without 1000 nM of EPZ compounds for 24 hours, and then stimulated with RANKL for 6 hours. Harvest cells for NF-κB luciferase assay. NF-κB Luciferase activity was measured by a luciferase reporter assay system (Promega) and a luminometer (SpectraMax i3x, Molecular Devices) following the manufacturer's manual.
